# Detection of SARS-CoV-2 in fecal samples with different pretreatment methods and PCR kits

**DOI:** 10.1186/s12866-021-02118-0

**Published:** 2021-02-19

**Authors:** Ranran Cao, Lirong Bao, Ming Pan, Cheng Zhang, Hongyu Liao, Li Liu, Yan Li, Mingyuan Li

**Affiliations:** 1 West China School of Basic Medical Sciences & Forensic Medicine, Sichuan University, Chengdu, 610041 China; 2 Sichuan Center for Disease Control and Prevention, Chengdu, 610041 China; 3 State Key Laboratory of Oral Diseases, National Clinical Research Center for Oral Diseases, West China Hospital of Stomatology, Sichuan University, Chengdu, 610041 China

**Keywords:** SARS-CoV-2, COVID-19, Fecal sample, Fecal–oral transmission, RT-PCR diagnostic kits

## Abstract

**Background:**

Gastrointestinal symptoms are common in COVID-19 patients and SARS-CoV-2 RNA has been detected in the patients’ feces, which could lead to fecal–oral transmission. Therefore, fecal sample testing with real-time RT-PCR is highly recommended as a routine test for SARS-CoV-2 infection. However, varying rates of detection in fecal sample have been reported. The aim of this study was to provide insights into the detection rates of SARS-CoV-2 in COVID-19 patients’ fecal sample by using four real-time RT-PCR kits and two pretreatment methods (inactive and non-inactive).

**Results:**

The detection rate of Trizol pretreatment group was slightly higher than that of Phosphate Buffered Saline (PBS) groups, showing that pretreatment and inactivation by Trizol had no influence to SARS-CoV-2 nucleic acid test (NAT) results. 39.29% detection rate in fecal sample by DAAN was obtained, while Bio-germ was 40.48%, Sansure 34.52%, and GeneoDx 33.33%. The former three kits had no significant difference. The DAAN kit detection rates of *ORF1ab* and *N* gene were nearly equal and Ct value distribution was more scattered, while the Bio-germ kit distribution was more clustered. The positive rate of SARS-COV-2 in fecal samples correlated with the severity of the disease, specifically, severe cases were less likely to be identified than asymptomatic infection in the DAAN group (adjusted OR 0.05, 95%CI = 0.00 ~ 0.91).

**Conclusions:**

Trizol should be of choice as a valid and safe method for pretreatment of fecal samples of SARS-CoV-2. All real-time RT-PCR kits assessed in this study can be used for routine detection of SARS-CoV-2 in fecal samples. While DAAN, with high NAT positive rate, could be the best out of the 4 kits used in this study. SARS-CoV-2 positive rate in fecal sample was related to the severity of illness.

**Supplementary Information:**

The online version contains supplementary material available at 10.1186/s12866-021-02118-0.

## Introduction

The Coronavirus Disease-19 (COVID-19), caused by severe acute respiratory syndrome coronavirus 2 (SARS-CoV-2), has resulted in an ongoing pandemic. Globally, as of 27 September 2020, there have been 32,730,945 confirmed cases of COVID-19, including 991,224 deaths [1]. Unlike other human coronaviruses, SARS-CoV-2 infects the lower respiratory tract and could lead to the severe respiratory symptoms. While the majority of infections lead to mild clinical manifestations [2, 3], such as cough, fever and malaise, some patients deteriorate to acute respiratory distress syndrome, multi-organ failure, shock, and blood clots, with high risk for a fatal outcome [2, 4, 5]. As SARS-CoV-2 invades cells through binding to angiotensin-converting enzyme 2 (ACE2) [6], which is expressed in various tissues apart from the lungs [7], COVID-19 could cause multiple organ involvements including gastrointestinal infection [8, 9], and fecal samples have tested positive for the nucleic acid of SARS-CoV-2, however, this could be simultaneous with respiratory tract samples testing negative [10]. Real-time reverse transcriptase-polymerase chain reaction (real-time RT-PCR) assay is one of the most sensitive and specific assay for viral nucleic acid test (NAT) and is widely recommended for the detection of SARS-CoV-2 [11, 12]. Different real-time RT-PCR positive rates in fecal samples have been reported by several studies [13–16], possibly due to different RNA isolation methods, or real-time RT-PCR kits. Coronaviruses belong to the Coronaviridae family in the Nidovirales order, with positive-sense RNA that can express its replication and transcription complex like RNA-dependent RNA polymerase (*RdRp*) from open reading frame 1ab (*ORF1ab*), a large open reading frame [17]. Coronavirus structural proteins, including envelope protein (E), nucleocapsid protein (N), and spike protein (S), are expressed by generating subgenomic messenger RNAs [18]. The *ORF1ab*, *N*, *RdRP*, *S* and *E* genes are the main targets of the viral RT-PCR detection protocols recommended by institutes worldwide [19].

The detection of SARS-CoV-2 in feces has recently aroused the concern about the potential fecal–oral or fecal–respiratory transmission route [16, 20–23]. Therefore, more precise detection of the virus in the feces is essential for understanding and preventing viral transmission. Nevertheless, fecal samples are far more complicated than nasopharyngeal/oropharyngeal swabs, sputum, and other respiratory samples, therefore it is critical to design an appropriate methodology to implement optimal sampling and RNA extraction procedures, for biases introduced from this process could influence detection.

In our study, fecal samples were collected from confirmed COVID-19 cases following the Guidelines supported by the National Health Commission of China (Version 7) [24]. Fecal samples were pretreated with Phosphate Buffered Saline (PBS) and Trizol, respectively. Few studies have assessed different kits for the NAT of SARS-CoV-2 in feces, so we screened four real-time RT-PCR kits in this study. Moreover, the demographical and clinical characteristics, severity of illness, fecal sample category, sampling interval and general information of kits used for viral NAT were compared between those cases. Our study provides evidence for the fecal positive rate of patients diagnosed with COVID-19 and effective selection of pretreatment methods and SARS-CoV-2 detection kits for fecal samples.

## Results

### Description of the population

For the 90 cases enrolled, median age was 46 years (interquartile range [IQR 35–59], range 7–83 years), with a balance gender ratio (M/F ratio, 42/48). Most patients were of moderate illness (56.67%), followed by severe illness (20.00%). Asymptomatic infection, mild, and critical patients were less than 10% each. Most fecal samples were collected 1 to 2 weeks after illness onset. The majority of fecal samples were categorized as formed stool, 6 cases of diarrhea were also collected. The patients covered a wide variety of careers, including peasant, unemployed or housewife (Table 1).

**Table 1:** Demographics and baseline characteristics of COVID-19 patients

	All patients (***n*** = 90)(%)
Age-median (IQR)	46 (35–59)
Age groups–No. (%)	
< 30 y	15 (16.67)
30–49 y	42 (46.67)
50–69 y	25 (27.78)
≥ 70 y	8 (8.89)
Gender–No. (%)	
Male	42 (46.67)
Female	48 (53.33)
Severity of illness–No. (%)	
Asymptomatic infection	6 (6.67)
Mild illness	9 (10.00)
Moderate illness	51 (56.67)
Severe illness	18 (20.00)
Critical illness	6 (6.67)
Days since the onset of illness to sampling–No. (%)	
≤ 7 days	23 (25.56)
7–14 days	43 (47.78)
> 14 days	22 (24.44)
Not known	2 (2.22)
Fecal sample category (Bristol Stool Scale)–No. (%)	
Anal swabs	10 (11.11)
Type 1–5	74 (82.22)
Type 6	3 (3.33)
Type 7	3 (3.33)
Occupation	
Peasant	22 (24.44)
Unemployed or housewife	14 (15.56)
Retirement	8 (8.89)
Student	8 (8.89)
Cadre	7 (7.78)
Business service	7 (7.78)
Worker	6 (6.67)
Teacher	4 (4.44)
Migrant worker	4 (4.44)
Medical staff	3 (3.33)
Others	7 (7.78)

*Abbreviations*: *COVID-19* Coronavirus Disease-19, *IQR* interquartile range

### Pretreatment with Trizol had no influence on NAT

Two pretreatment methods were used to suspend 38 fecal samples. The double positive rates of PBS, Trizol groups were 55.26% (21/38) and 60.53% (23/38), respectively, when using Bio-germ Kit. The double positive rates of GeneoDx were 39.47% (15/38) and 44.74% (17/38), respectively. Paired chi-square test was used for analysis, and it was found that there was no statistical difference in the positive rate of the same kit using different pretreatment methods (*P* > 0.05), and there was no statistical difference between the two kits. However, the detection rate of Trizol group (60.53, 44.74%) was slightly higher than that of PBS group.

### The positive rate of SARS-CoV-2 in fecal samples

The positive rate by four real-time RT-PCR kits with the fecal samples pretreated by Trizol ranged from 33 to 40%, with DAAN (33/84, 39.29%) and Bio-germ (34/84, 40.48%) performing better, followed by Sansure (29/84, 34.52%) and GeneoDx (22/66, 33.33%) (Table 2). McNemar’s exact test found statistically significant differences between DAAN and GeneoDx (*P* < 0.05) and between Bio-germ and GeneoDx (*P* < 0.05), while other pairwise comparisons showed no statistically significant differences.

**Table 2:** The positive rate of SARS-CoV-2 in fecal samples

Kits	***N*** positive (%)	***ORF1ab*** positive (%)	Double positive (%)
DAAN (*n* = 84)	33 (39.29)	35 (41.67)	33 (39.29)^a^
Sansure (*n* = 84)	52 (61.90)	29 (34.52)	29 (34.52)
Bio-germ (*n* = 84)	38 (45.24)	35 (41.67)	34 (40.48)^b^
GeneoDx (*n* = 66)	28 (42.42)	22 (33.33)	22 (33.33)^a,b^

*Abbreviations*: *ORF1ab* open reading frame 1ab of SARS-CoV-2, *SARS-CoV-2* severe acute respiratory syndrome coronavirus 2

^a^*P* < 0.05, DAAN VS. GeneoDx

^b^*P* < 0.05, Bio-germ VS. GeneoDx

**Fig. 1:**
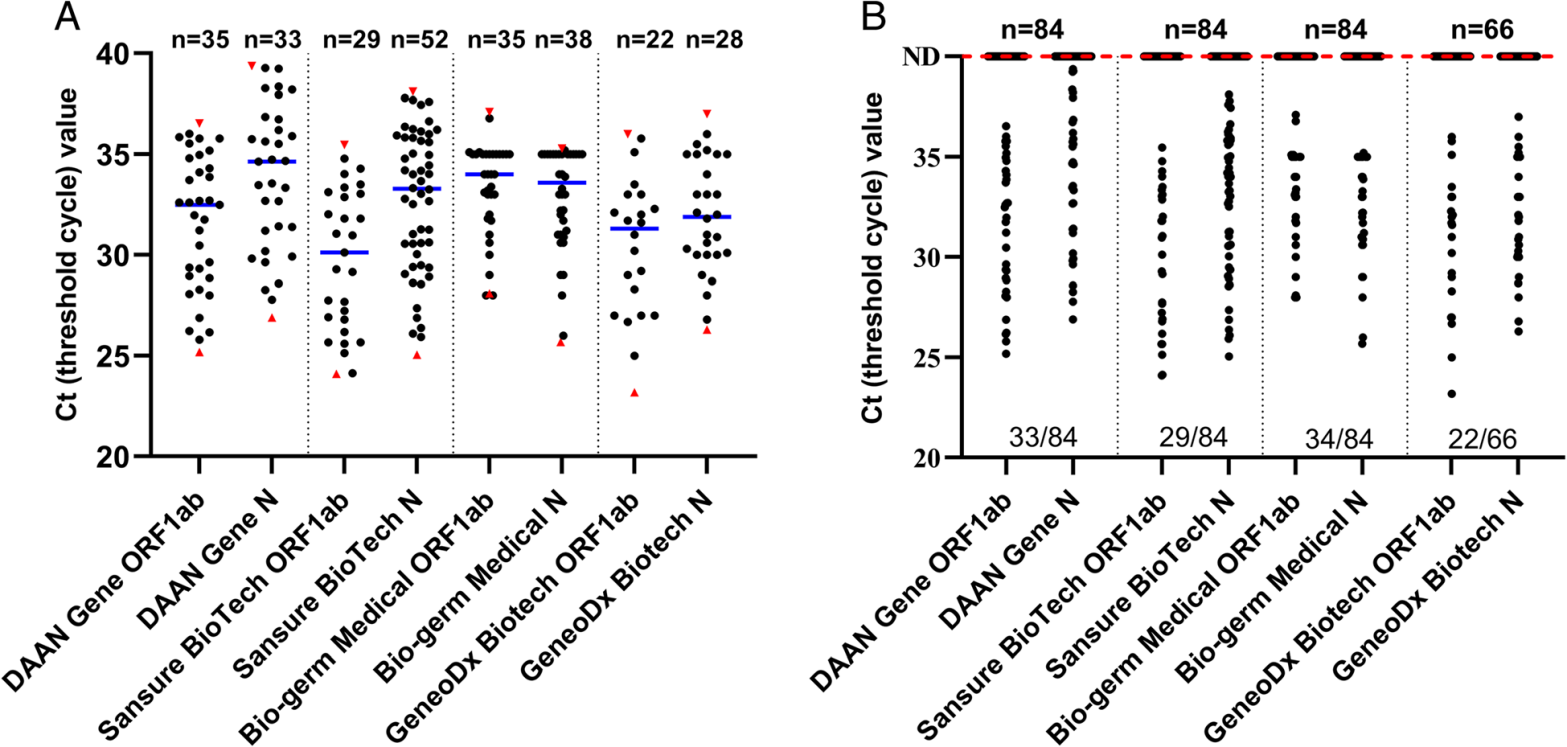
Different kits showed variations in detection rate and Ct values in Trizol group. Graph depicts Ct values obtained for all clinical samples in all RT-PCR assays. **a** Ct values of *ORF1ab* and *N* genes detected by the 4 kits. **b** Data points above the horizontal dotted line are negative. The detection rate of the complete real-time RT-PCR kit is indicated below the data points, e.g. 33/84 means 33 out of 84 samples tested positive. The blue lines show the mean Ct value for each assay, triangles show the Ct values of the samples with the highest and lowest concentration. N, nucleocapsid protein of SARS-CoV-2. *ORF1ab*: open reading frame 1ab of SARS-CoV-2; RT-PCR: reverse transcriptase-polymerase chain reaction; SARS-CoV-2: severe acute respiratory syndrome coronavirus 2

The Ct values of *ORF1ab* and *N* genes detected by the 4 kits were different to some extent (Fig. 1a). The positive numbers of *ORF1ab* and *N* gene detected with DAAN kit were nearly equal, i.e., 35 and 33, respectively. Followed by, Bio-germ, 36 and 40, respectively. The double positive rate of these two kits was also the highest (39.29, 40.48%), and the Ct value distribution of Bio-germ was more clusterred, while that of DAAN was more scattered. The Ct values of *ORF1ab* gene ranged from 28 to 37.1, and *N* gene from 25.7 to 35.3 in Bio-germ group. The Ct values of *ORF1ab* gene ranged from 25.19 to 36.55, and *N* gene from 26.91 to 39.37 in DAAN group. Further research is needed concerning the sensitivity. In addition, Sansure showed the largest number difference between the two genes, with 29 *ORF1ab* gene positive samples and 52 *N* gene positive samples. The Ct values of *ORF1ab* and *N* genes double positive is shown in Fig. 1b. Interestingly, the positive rate of Bio-germ was still higher (34/84) than that of GeneoDx (22/66) after enlarging the sample size.

### The positive rate of SARS-CoV-2 correlates with the severity of the disease

When using kits from DAAN and Bio-germ, the positive rates of different clinical types were statistically significant (Fisher’s Exact test, two-tailed, *P* < 0.05) (Additional file 1: Supplementary Table 1). Univariate logistic regression models were used to test the correlation between individual covariates and the outcome of a positive test, and multivariate logistic regression was used to detect variables that were associated with a positive test for SARS-CoV-2 after multiple imputations of missing values. In univariable analysis, there was no significant association between NAT positive rate and severity of illness. Moreover, in multivariable analysis, after adjusting for age, gender and fecal samples category, it was found that fecal samples from severe cases were less likely to be identified than asymptomatic infection when using the DAAN kit (adjusted odds ratio (OR) 0.05, 95%confidence interval (CI) = 0.00–0.91) (Table 3). While a similar correlation was not observed in the Bio-germ kit group (Additional file 1: Supplementary Table 2). This may have been due to the small sample size and the small number of grids in some clinical classifications, which makes it hard to calculate the risk coefficient or the large confidence interval.

**Table 3:** Risks of SARS-CoV-2 positive rate upon severity of illness (DAAN)

Risk factors	Positive (***n*** = 33)(%)	Negative (***n*** = 51) (%)	Unadjusted OR (95%CI)	***P***-value	Adjusted OR^**a**^ (95%CI)	***P***-value
Asymptomatic infection	4 (12.12)	1 (1.96)	1		1	–
Mild	0 (0.00)	8 (15.69)	–	–	–	–
Moderate	20 (60.61)	27 (52.94)	0.19 (0.02–1.79)	0.145	0.13 (0.10–1.60)	0.111
Severe	5 (15.15)	13 (25.49)	0.10 (0.01–1.08)	0.058	0.05 (0.00–0.91)	0.043^b^
Critical	4 (12.12)	2 (3.92)	0.50 (0.03–7.99)	0.624	0.25 (0.01–5.71)	0.386

*Abbreviations*: *CI* confidence interval, *OR* odds ratio, *SARS-CoV-2* severe acute respiratory syndrome coronavirus 2

^a^Adjusted age, gender and fecal samples category

^b^*P* < 0.05

### There was no correlation between sampling interval and positive results

Logistic regression models were used to test the correlation between sampling interval and the outcome of a positive test. The sampling interval was defined as the days from illness onset to the sampling time (≤ 7 d, 7–14 d and > 14 d). We attempted to analyze NAT results from all the kits and adjusted for age, gender and fecal samples category, but no obvious differences were found in our results (Additional file 1: Supplementary Tables 3–6).

## Discussion

With the rapid spread of the SARS-CoV-2, there have been a large number of research studies on the detection of the viral NAT, such as the positive detection rate in different clinical samples [16, 25], comparison of clinical performance of different real-time RT-PCR kits [26], etc. But few articles mentioned the process of fecal samples pretreatment and the impact of different pretreatment methods on NAT. The composition of fecal samples is more complex than that of respiratory samples and there are many impurities, hence proper pretreatment must be performed before viral RNA isolation and purification. At present, the method of vortexing with NS or PBS to prepare a feces suspension followed by centrifuging to create a supernatant for RNA extraction is typically adopted. However, the infectivity, transmission ability, and survivability of this novel virus remain unclear. Vortexing and centrifuging are high-risk biohazardous operations which produce laboratory aerosols. Trizol is a common RNA extraction reagent, comprised of phenol and guanidine isothiocyanate, can lyse cells, ensure RNA integrity, and has a strong protein denaturation effect. Some studies have proven that Trizol could inactive high-titer viruses, including SARS-CoV-2 [27, 28]. Therefore, Trizol can not only extract RNA effectively and ensure the integrity of RNA, but also inactivate virus, reducing infectivity and ensure biosafety. In our study, PBS and Trizol pretreatments were compared. The detection rate of the Trizol group was slightly higher than that of PBS group, though there was no statistical difference. So we concluded that fecal samples treated with Trizol can not only inactive the virus and reduce the risk of the experimental procedure, but also had no influence on the downstream NAT. Trizol is a monophasic solution which solubilizes biological material and denatures protein simultaneously [29]. Trizol can be recommended to treat fecal samples to help improve the sensitivity with low limit of detection, and maintain viral RNA integrity, and samples could be inactivated in Trizol, thus reduce infectivity and the chance of exposing healthcare workers [30]. However, further research is required before Trizol could be used as a routine reagent in laboratory protocols related to SARS-CoV-2.

The positive rates of SARS-CoV-2 in fecal samples of COVID-19 patients testing by real-time RT-PCR ranged from 25 to 82% [10, 16, 22, 31–35]. An astonishing study reported that 39 (53.42%) out of the 73 hospitalized COVID-19 patients’ fecal samples were tested SARS-CoV-2 positive and remained positive for 1–12 days, 17 (23.29%) of whom persisted even after the respiratory samples turned negative for the viral detection [22]. In our study, all the detection rates in feces were more than 30% (DAAN 39.29%, Sansure 34.52%, Bio-germ 40.48%, GeneoDx 33.33%). The difference of detection rates of SARS-CoV-2 in fecal samples may result from different sample sizes, different kits and the various and unstandardized sampling time. Accumulating evidence has supported the potential for feces associated transmission of SARS-CoV-2 [8, 10, 16, 22]. By evaluating the positive detection rate of the four kits, the distribution of Ct values, and the positive numbers of *N* and *ORF1ab* genes, we recommend the use of DAAN for NAT of fecal samples.

Other research groups have found co-expression of ACE2 and transmembrane serine protease 2 (TMPRSS2) in the enterocytes, progenitor, and stem-like epithelial cells of the lower gastrointestinal tract, especially in the small intestine [36]. It is well established that the invasion of SARS-CoV-2 depends on the interaction of spike protein with ACE2 and TMPRSS2 [6, 37, 38]. In addition, staining of ACE2 and SARS-CoV-2 was simultaneously observed in gastrointestinal epithelium from those who tested positive for SARS-CoV-2 in fecal samples [22]. Surprisingly, a recent report showed that the toilet bowl, sink, and door handle in the room where the COVID-19 patient had resided were contaminated by SARS-CoV-2 [39]. This revealed that the SARS-CoV-2 can transmit through feces as well as respiratory droplets or contact transmission. Therefore, we believe, prevention of fecal–oral transmission should be considered, and all COVID-19 patients’ fecal samples should be as a routine test for the SARS-CoV-2. Once it is positive, the special regulations and nursing strategies must be used to prevent spread of the virus. Further research is needed to understand to what extent SARS-CoV-2 is transmitted via fecal–oral route. This will help decide whether to test the virus in the feces of COVID-19 patients.

## Methods

### Fecal sample collection

A total of 90 fecal samples of confirmed COVID-19 cases in Sichuan Province, China were collected and stored in sterile containers at − 80 °C.

### Pretreatment for the fecal samples and RNA extraction

To study the influence of different pretreatment methods on SARS-CoV-2 NAT results, 38 fecal samples were selected randomly to suspend in two ways. To be specific, 200 mg of stool was suspended in a 15 mL tube containing 2 mL PBS, 2 mL Trizol (Trizol^@^ Reagent, invitrogen, USA), respectively. The mixture was stirred gently and mixed. Keep still for 10 min and then supernatant were transferred to a new tube. Depending on the NAT results of the 38 randomly selected fecal samples, the remaining 52 samples were suspended in Trizol. 200 μL supernatant were used for RNA isolation by the NP968 Nucleic Acid Extraction System (Xi’an Tianlong Science & Technology Co., LTD, Xi’an, China). Extracted RNA was stored at − 20 °C until use.

### Real-time RT-PCR assays for the detection of SARS-CoV-2

#### Group 1

To find the best pretreatment method, the viral RNA extracted from 38 fecal samples suspend in two ways were tested with two kits (Shanghai Bio-germ Medical Co., Ltd., and Shanghai GeneoDx Biotech Co., Ltd), respectively.

#### Group 2

To study positive rate of SARS-CoV-2 in fecal samples and evaluate real time RT-PCR kits, the viral RNA extracted from 84 fecal samples suspend in Trizol were tested with four kits (Shanghai Bio-germ Medical Co., Ltd., and Shanghai GeneoDx Biotech Co., Ltd. DAAN Gene Co., Ltd. of Sun Yat-sen University and Sansure Biotech Inc.), respectively. As 6 stool samples did not contain enough material for RNA extraction, only 84 samples were tested in group 2. Due to a supply shortage, only 66 fecal samples in group 2 were tested with the GeneoDx kit. As previously described [40], only when the two target genes (*ORF1ab and N*) are simultaneously positive, can a positive detection of SARS-CoV-2 be reported.

### Characteristics of the selected kits

Here, we provided a comparison of four readily available COVID-19 real-time RT-PCR kits from different manufacturers (Table 4). Two of these kits have been proven to detect low concentration viral RNA: Sansure can achieve 1 copy/reaction, and Bio-germ can achieve 10 Copies/reaction [41]. The 4 commercial kits have been approved by the China National Medical Products Administration (NMPA), and 2 have received CE (Conformité Européenne) marking (DAAN and Sansure). All real-time RT-PCR kits assessed in this study have been used for routine diagnostics of SARS-CoV-2 by experienced molecular diagnostic laboratories.

**Table 4:** General information of kits for detection of SARS-COV-2 by real-time RT-PCR

Kit^a^	Catalog No.	Registration No.	Storage temperature	RNA template (μL)	PCR system (μL)	Target genes	Positive judgment Ct value
DAAN	DA0930-DA0932	20,203,400,063	−20 ± 5 °C	5	25	*ORF1ab*/*N*	≤ 38
Sansure	S3103E	20,203,400,064	−20 ± 5 °C	20	50	*ORF1ab*/*N*	≤ 40
Bio-germ	SJ-HX-226-1, 2	20,203,400,065	−70 °C	5	25	*ORF1ab*/*N*	≤ 38
GeneoDx	GZ-D2RM25	20,203,400,058	−20 ± 5 °C	2	20	*ORF1ab*/*N*	< 40

*Abbreviations*: *ORF1ab* open reading frame 1ab of SARS-CoV-2, *PCR* polymerase chain reaction, *SARS-CoV-2* severe acute respiratory syndrome coronavirus 2

^a^The web links for the 4 approved kits are DAAN, http://en.daangene.com/; Sansure, http://eng.sansure.com.cn/; and Bio-germ, http://bio-germ.com/; GeneoDx, http://www.geneodx.com/

### Data analysis

Statistical analyses were performed by using SPSS26.0 software. All statistical tests were two-sided, and significant differences were considered at *P* < 0.05. Continuous variables were evaluated using the median and interquartile range (IQR) values. Chi-square or Fisher’s exact tests were utilized to compare the proportions of the categorical variables. Chi-square test was used to compare inter-group differences, and logistic regression analysis was performed to analyze the risk factors for SARS-CoV-2 prevalence. Crude and adjusted risk ratios and 95%CIs for NAT positive rate were calculated using modified Poisson regression. Risk ratios were adjusted for age (as a continuous variable), gender, and fecal samples category.

## Supplementary Information

**Additional file 1 Supplementary Table 1.** Fisher’s Exact test for the positive rate of confirmed cases of different clinical types. **Supplementary Table 2.** Risks of SARS-CoV-2 positive rate upon severity of illness (Bio-germ). **Supplementary Table 3.** Logistic regression analysis of Sampling intervals and DAAN testing results. **Supplementary Table 4.** Logistic regression analysis of Sampling intervals and Sansure testing results. **Supplementary Table 5.** Logistic regression analysis of Sampling intervals and Bio-germ testing results. **Supplementary Table 6.** Logistic regression analysis of Sampling intervals and GeneoDx testing results.

## Data Availability

All data generated or analyzed during this study are included in this published article and its supplementary information files.

## Abbreviations

ACE2: Angiotensin-converting enzyme 2
CE: Conformité Européenne
CI: Confidence interval
COVID-19: Coronavirus Disease-19
IQR: Interquartile range
NAT: Nucleic acid detection
NMPA: National Medical Products Administration
OR: Odds ratio
*ORF1ab*: Open reading frame 1ab of SARS-CoV-2
PBS: Phosphate Buffered Saline
*RdRp*: RNA-dependent RNA polymerase
RT-PCR: Reverse transcriptase-polymerase chain reaction
SARS-CoV-2: Severe acute respiratory syndrome coronavirus 2
TMPRSS2: Transmembrane serine protease 2
