# Supplementary material for: Detection of SARS-CoV-2 in fecal samples with different pretreatment methods and PCR kits
Source: BMC Microbiol. 2021 Feb 19;21:56. doi: 10.1186/s12866-021-02118-0 (PMC7893130; doi:10.1186/s12866-021-02118-0)
Supplement: Supplementary file 1 — Additional file 1 Supplementary Table 1. Fisher’s Exact test for the positive rate of confirmed cases of different clinical types. Supplementary Table 2. Risks of SARS-CoV-2 positive rate upon severity of illness (Bio-germ). Supplementary Table 3. Logistic regression analysis of Sampling intervals and DAAN testing results. Supplementary Table 4. Logistic regression analysis of Sampling intervals and Sansure testing results. Supplementary Table 5. Logistic regression analysis of Sampling intervals and Bio-germ testing results. Supplementary Table 6. Logistic regression analysis of Sampling intervals and GeneoDx testing results. [file 12866_2021_2118_MOESM1_ESM.doc]

**Additional file 1**

**Supplementary Tables**

**Detection of SARS-CoV-2 in fecal samples with different pretreatment methods and PCR kits**

**Supplementary Table 1** Fisher’s exact test for the positive rate of confirmed cases of different clinical types

|  | DAAN | Sansure | Bio-germ | GeneoDx |
| --- | --- | --- | --- | --- |
| *n* | 84 | 84 | 90 | 66 |
| Fisher’s Exact test | 11.745 | 2.882 | 9.051 | 7.329 |
| *P*-value | 0.013^a^ | 0.605 | 0.049^a^ | 0.098 |

^a^*P*<0.05

**Supplementary Table 2** Risks of SARS-CoV-2 positive rate upon severity of illness (Bio-germ)

|  | Positive (*n*=33)(%) | Negative (*n*=51)(%) | Unadjusted OR (95%CI) | *P*-value | Adjusted OR^a^(95%CI) | *P*-value |
| --- | --- | --- | --- | --- | --- | --- |
| Asymptomatic infection | 3 (8.57) | 3 (5.45) | 1.00 |  |  |  |
| Mild illness | 4 (11.43) | 5 (9.09) | 0.63 (0.06–6.96) | 0.705 | 0.80 (0.10–6.35) | 0.833 |
| Moderate illness | 20 (57.14) | 31 (56.36) | 0.43 (0.06–3.02) | 0.396 | 0.65 (0.12–3.52) | 0.613 |
| Severe illness | 3 (8.57) | 15 (27.27) | 0.11 (0.01–1.2) | 0.07 | 0.20 (0.03–1.51) | 0.119 |
| Critical illness | 5 (14.29) | 1 (1.82) | 2.56 (0.14–48.4) | 0.532 | 5.00 (0.34–72.77) | 0.239 |

*Abbreviations*: *CI* confidence interval, *OR* odds ratio, *SARS-CoV-2*severe acute respiratory syndrome coronavirus 2

^a^Adjusted age, gender and fecal samples category

**Supplementary Table 3** Logistic regression analysis of sampling intervals and DAAN testing results

|  | Positive (*n*=33) (%) | Negative (*n*=51) (%) | Unadjusted OR (95%CI) | *P*-value | Adjusted OR^a^(95%CI) | *P*-value |
| --- | --- | --- | --- | --- | --- | --- |
| Sampling intervals |  |  |  |  |  |  |
| ≤7 d | 12 (36.36) | 11 (21.57) | 1.00 | - | 1.00 | - |
| 7–14 d | 14 (42.42) | 27 (52.94) | 0.48 (0.17–1.35) | 0.162 | 0.40 (0.13–1.23) | 0.11 |
| >14 d | 7 (21.21) | 13 (25.49) | 0.49 (0.14–1.69) | 0.261 | 0.37 (0.09–1.41) | 0.14 |

*Abbreviations*: *CI* confidence interval, *OR* odds ratio

^a^Adjusted age, gender and fecal samples category

**Supplementary Table 4** Logistic regression analysis of sampling intervals and Sansuretesting results

|  | Positive (*n*=29) (%) | Negative (*n*=55) (%) | Unadjusted OR (95%CI) | *P*-value | Adjusted OR^a^(95%CI) | *P*-value |
| --- | --- | --- | --- | --- | --- | --- |
| Sampling intervals |  |  |  |  |  |  |
| ≤7 d | 10 (34.48) | 13 (23.64) | 1.00 | - | 1.00 | - |
| 7–14 d | 11 (37.93) | 30 (54.55) | 0.48 (0.16–1.4) | 0.177 | 0.39 (0.12–1.21) | 0.101 |
| >14 d | 8 (27.59) | 12 (21.82) | 0.87 (0.26–2.93) | 0.818 | 0.97 (0.27–3.52) | 0.961 |

*Abbreviations*: *CI* confidence interval, *OR* odds ratio

^a^Adjusted age, gender and fecal samples category

**Supplementary Table 5** Logistic regression analysis of sampling intervals and Bio-germ testing results

|  | Positive (*n*=35) (%) | Negative (*n*=55) (%) | Unadjusted OR (95%CI) | *P*-value | Adjusted OR^a^(95%CI) | *P*-value |
| --- | --- | --- | --- | --- | --- | --- |
| Sampling intervals |  |  |  |  |  |  |
| ≤7 d | 11 (31.43) | 12 (21.82) | 1.00 | - | 1.00 | - |
| 7–14 d | 17 (48.57) | 28 (50.91) | 0.66 (0.24–1.83) | 0.427 | 0.72 (0.25–2.07) | 0.545 |
| >14 d | 7 (20.00) | 15 (27.27) | 0.51 (0.15–1.71) | 0.276 | 0.49 (0.14–1.76) | 0.272 |

*Abbreviations*: *CI* confidence interval, *OR* odds ratio

^a^Adjusted age, gender and fecal samples category

**Supplementary Table 6** Logistic regression analysis of sampling intervals and GeneoDx testing results

|  | Positive (*n*=22) (%) | Negative (*n*=44) (%) | Unadjusted OR (95%CI) | *P*-value | Adjusted OR^a^(95%CI) | *P*-value |
| --- | --- | --- | --- | --- | --- | --- |
| Sampling intervals |  |  |  |  |  |  |
| ≤7 d | 6 (27.27) | 16 (36.36) | 1.00 | - | 1.00 | - |
| 7–14 d | 10 (45.45) | 20 (45.45) | 1.33 (0.40–4.46) | 0.640 | 1.66 (0.45–6.05) | 0.445 |
| >14 d | 6 (27.27) | 8 (18.18) | 2.00 (0.49–8.23) | 0.337 | 2.57 (0.55–12.09) | 0.231 |

*Abbreviations*: *CI* confidence interval, *OR* odds ratio

^a^Adjusted age, gender and fecal samples category
